# Machine Learning and Feature Selection Methods for Disease Classification With Application to Lung Cancer Screening Image Data

**DOI:** 10.3389/fonc.2019.01393

**Published:** 2019-12-11

**Authors:** Darcie A. P. Delzell, Sara Magnuson, Tabitha Peter, Michelle Smith, Brian J. Smith

**Affiliations:** ^1^Department of Mathematics and Computer Science, Wheaton College, Wheaton, IL, United States; ^2^Department of Biostatistics, University of Iowa, Iowa City, IA, United States

**Keywords:** radiomics, machine learning, CT image, biomarkers, lung cancer

## Abstract

As awareness of the habits and risks associated with lung cancer has increased, so has the interest in promoting and improving upon lung cancer screening procedures. Recent research demonstrates the benefits of lung cancer screening; the National Lung Screening Trial (NLST) found as its primary result that preventative screening significantly decreases the death rate for patients battling lung cancer. However, it was also noted that the false positive rate was very high (>94%).In this work, we investigated the ability of various machine learning classifiers to accurately predict lung cancer nodule status while also considering the associated false positive rate. We utilized 416 quantitative imaging biomarkers taken from CT scans of lung nodules from 200 patients, where the nodules had been verified as cancerous or benign. These imaging biomarkers were created from both nodule and parenchymal tissue. A variety of linear, nonlinear, and ensemble predictive classifying models, along with several feature selection methods, were used to classify the binary outcome of malignant or benign status. Elastic net and support vector machine, combined with either a linear combination or correlation feature selection method, were some of the best-performing classifiers (average cross-validation AUC near 0.72 for these models), while random forest and bagged trees were the worst performing classifiers (AUC near 0.60). For the best performing models, the false positive rate was near 30%, notably lower than that reported in the NLST.The use of radiomic biomarkers with machine learning methods are a promising diagnostic tool for tumor classification. The have the potential to provide good classification and simultaneously reduce the false positive rate.

## 1. Introduction

Publication of primary results from the National Lung Screening Trial (NLST) reported that lung cancer screening, especially when performed with low dose computed tomography (CT) scans, can significantly reduce the mortality rate of lung cancer. This result highlights the benefits of lung cancer screening; however, the NLST also found that screening results had a notably high rate of false positive results. Of the total number of low dose CT scans in the NLST, the false positive rate surpassed 94% (1). The NLST researchers noted that the high false positive rate was a challenge which required further research, and that challenge persists to the present. The negative consequences associated with false positive exam results can include patient anxiety and unnecessary invasive diagnostic procedures such as biopsy (2, 3).

High-throughput extraction of features from imaging data composes the essence of radiomics, an emerging field of research which offers significant improvement to decision-support in oncology (4, 5). Current work examines the predictive power of quantitative imaging biomarkers, which are quantitative features extracted from routine medical images (4, 6, 7), as inputs within predictive classifying models. The information contained in the imaging biomarkers has the potential to improve classification accuracy in a variety of statistical models (2).

Across the literature, quantitative biomarkers taken from imaging data have been used to develop models with the intent to identify and analyze associations between radiomic/nodule features (stages or histological characteristics) and clinical outcomes (survival, recurrence, etc.). Previous work in radiomics aimed at classification of lung nodules has examined a variety of outcomes (5, 8–12). Zhu et al. used outcome categories for lung cancer type with a LASSO classification model (13). Zhang et al. examined outcomes for local/distant failure using several machine learning classifiers (5). Pamar et al. used clusters of biomarkers as predictors in models of overall survival (14). Dilger et al. used an expanded set of radiomic features that included both nodule and parenchymal tissue. They showed an increase in classification performance when the parenchymal tissue was included in feature extraction (3).

In this paper, we investigate the predictive power of biomarkers (computed from both nodule and parenchymal tissue as calculated by Dilger et al. (3)) to classify lung nodule status as malignant/benign while also considering the false positive rate. Our comprehensive approach includes multiple combinations of models and filtering techniques. In particular, combinations of twelve machine learning classifiers along with six feature selection methods were compared, using area under the receiver operating characteristic curve (AUC) as the model performance metric.

## 2. Methods

### 2.1. Dataset

This retrospective study analyzed data originally taken from 200 CT scans of the lungs of patients at the University of Iowa Hospital. Pathology and radiology reports were reviewed to identify an analysis set of patients who met eligibility criteria of having (a) a solitary lung nodule (5–30 mm) and (b) a malignant nodule confirmed on histopathology or a benign nodule confirmed on histopathology or by size stability for at least 24 months. Manual segmentations were performed by a graduate student trained in medical image analysis in order to define a region of interest (ROI) around each nodule. The ROIs were defined to include amounts of parenchyma approximately proportional to the nodule sizes. Individual ROI voxels were labeled as belonging to either the nodule or the parenchyma, with radiomic features calculated separately for each to produce the complete set of 416 (approximately half nodule and half parenchyma) quantitative imaging biomarkers. These biomarkers measured features such as intensity, shape, and texture of the ROI (15). This study is a secondary analysis of de-identified data originally collected with approval from the University of Iowa institutional review board. Demographic information can be found in Table 1.

**Table 1:** Demographics of patient cohort.

	**Malignant**	**Benign**
Number of patients	110	90
Female	51 (46.4%)	63 (70.0%)
Male	59 (53.6%)	27 (30.0%)
Age, yrs (mean ±SD)	65.7 ± 11.2	58.2 ± 13.2
Pack-years (mean ±SD)	38.4 ± 31.2	11.2 ± 16.9
Nodule size, mm (range, mean ±SD)	7−44, 19.1 ± 6.3	6−30, 15.2 ± 5.8

A strength of the dataset is its fairly balanced malignant/ benign status breakdown, with 45% of the cases malignant and 55% benign. Many machine learning-based classifying algorithms assume that the outcomes of a data set are balanced, but this assumption is not met when the proportion of outcomes is highly uneven. The data set used in this work has a nearly even ratio of malignant and benign nodules (16).

### 2.2. Radiomic Features

The 416 radiomic features which were available for this investigation quantified nodule characteristics from CT images acquired from a variety of scanner protocols through the University of Iowa Hospital. The most common CT models used were Siemens SOMATOM Definition, Siemens Sensation 16, Sensation Biograph 40, and Toshiba Aquilion. Using these machines, several protocols were used, including Chest CT scans with and without contrast, CT Angiography scans, Extrenal CT scans, PET/CT scans, and CT: Chest, Abdomen, and Pelvis scans. Slice thickness ranged from 1.0 to 6.0 mm with an average of 3.3 mm (15). From these scans, voxels labeled as parenchyma and nodule were used in the extraction of four classes of features: intensity, shape, border, and texture. The intensity of CT images described the radiodensity of the anatomy [measured using Hounsfield units (HU)] as well as heterogeneity of the nodule. Shape features examined sphericity and the maximum diameter of the nodule. Sphericity was computed by comparing the volume of the nodule to its surface area, and maximum diameter was measured using the Response Evaluation Criteria in Solid Tumors (RECIST). The border features were measured using a rubber band straightening transform (RBST). The texture features were extracted from the nodule and parenchyma regions using Laws' Texture Energy Measures (TEM). From these TEMs, the mean, variance, kurtosis, and skewness of the nodule and parenchyma were extracted. Radiomic features were extracted using a Matlab based CAD tool, and the mathematical definitions for all of the radiomic measurements are described in full in Dilger (17).

### 2.3. Feature Selection Methods

As is common in radiomics studies with hundreds of features, many of the biomarkers (features) used as predictors were highly correlated with one another; this challenge necessitated feature selection in order to avoid collinearity, reduce dimensionality, and minimize noise (11, 16, 18, 19). To this end, we considered three feature selection methods: a linear combinations filter, a pairwise correlation filter, and principle component analysis.

For the linear combinations filter (lincom), a QR decomposition along with an iterative procedure is used to determine if some predictors are linear combinations of others. Predictors are sequentially removed until the design matrix is full rank. The pairwise correlation filter removes those predictors whose pairwise correlation is greater than a specified cutoff. The two predictors with the largest absolute correlation are first considered. Of those two, the predictor with the highest average absolute correlation with all other variables is removed. This process continues until all the predictors left have pairwise absolute correlations less than the cutoff. After investigating multiple cutoffs, we chose a cutoff value of 0.95 for the pairwise correlation filter (corr.95) since this cutoff removed highly correlated variables but still retained a large number of features. Principal component analysis reduces dimensionality by creating new, uncorrelated predictors which explain a large proportion of the variance in the predictor space. Principal component analysis was implemented at three different cutoffs (pca.85, pca.90, pca.95), where the number of components accounted for either 85, 90, or 95% of the variance in the predictor space (Table 2).

**Table 2:** Summary of feature selection methods.

**Feature selection method**	**Abbreviation**
Linear combination	lincom
Pairwise correlation	corr.95
PCA - 0.85 cutoff	pca.85
PCA - 0.90 cutoff	pca.90
PCA - 0.95 cutoff	pca.95
Unfiltered	nofilter

### 2.4. Classifiers and Performance Metrics

Combinations of the six feature selection methods and twelve classifiers were investigated by implementing a 10-fold repeated cross-validation framework with five repeats, a standard validation technique (5, 13, 16, 20, 21). The feature selection methods were included in the cross-validation algorithm so that their contribution to the final model fit is reflected in the performance metrics. The classifiers are from three different families: linear, nonlinear, and ensemble (22). Of the linear classifiers, an elastic net (elasticnet), a logistic regression (logistic), a partial least squares model (pls), and a logistic regression with Step AIC were fit. The nonlinear classifiers include a K-nearest neighbors model (knn), a neural network (nnet), and three support vector machines: a linear kernel (svml), a polynomial kernel (svmpoly), and a radial kernel (svmr). The ensemble models used included bagged classification trees (bag), random forest (rf), and stochastic gradient boosting (gbm) (Table 3).

**Table 3:** Summary of classifiers.

**Model family**	**Classifier**	**Abbreviation**
	Elastic net	elasticnet
Linear	Logistic regression	logistic
	Partial least squares	pls
	Logistic regression with step AIC	glmStepAIC
	K-nearest neighbors	knn
	Neural network	nnet
Nonlinear	Support vector machine (linear kernel)	svml
	SVM (polynomial kernel)	svmpoly
	SVM (radial kernel)	svmr
	Bagged trees	bag
Ensemble	Random forest	rf
	Stochastic gradient boosting	gbm

The quality of model performance in most machine learning algorithms is dependent upon the choice of various tuning parameters. Some tuning parameters take into account the number of predictors after feature selection. For example, the mtry tuning parameter for rf, which determines the number of candidate variables at each branch, is equal to the square root of the number of predictors. Other tuning parameters were chosen based on standard practice (22, 23). For example, the decay tuning parameter for nnet, which helps prevent overfitting, generally takes the values of 0.1, 0.01, and 0.001. All models were fit using the caret R package (24). Our R code implementing the feature selection and classification models is presented as Supplementary Material.

## 3. Results

The linear combinations filter removed 217 biomarkers, leaving a set of 199 predictors. The pairwise correlation filter retained 39 predictors, while principal components analysis retained 12, 14, and 18 components at the 85, 90, and 95% levels, respectively.

**Figure 1:**
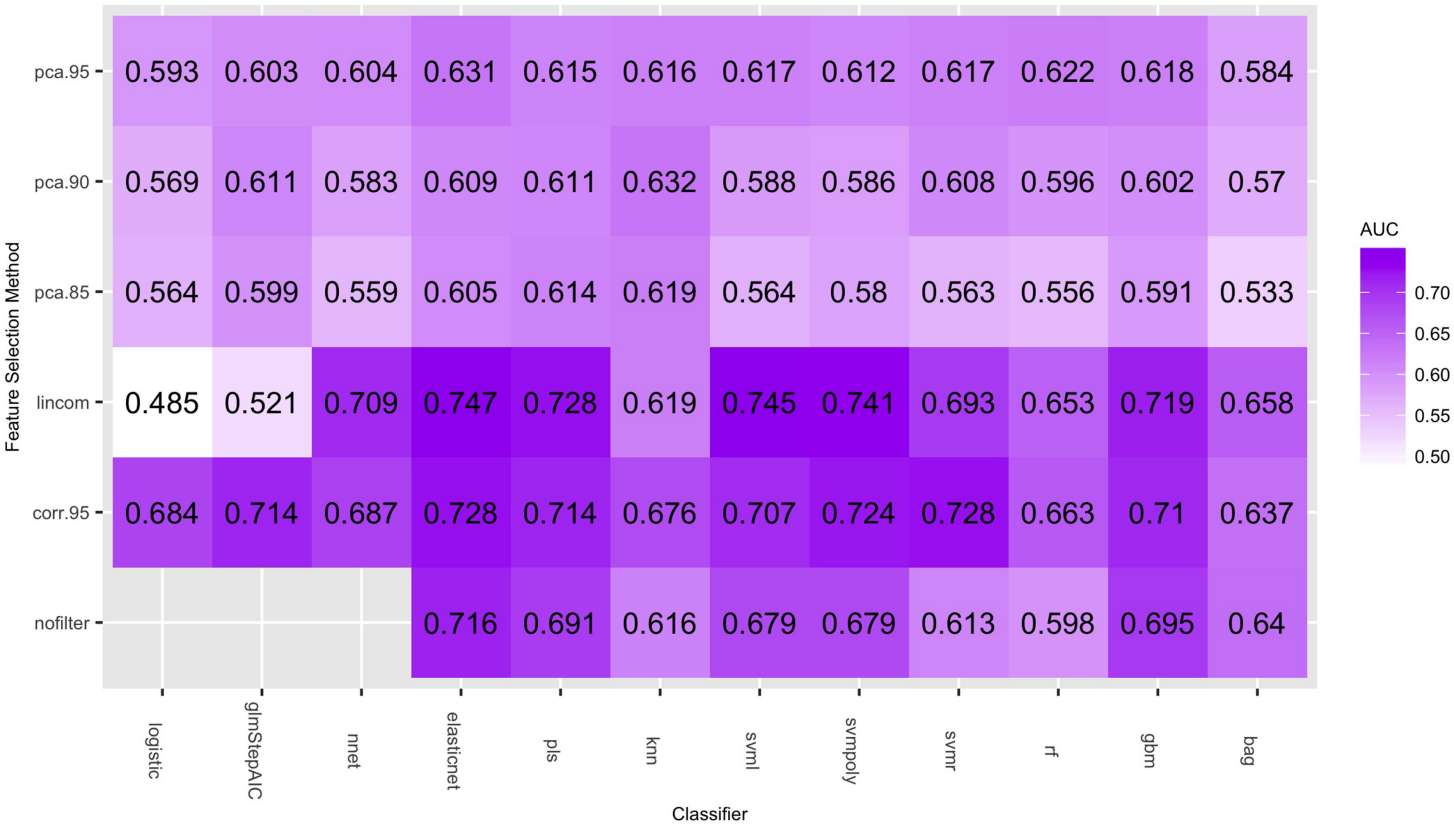
Average AUC values (over the 50 repeated cross-validation testing sets) of each feature selection/classifier combination.

Figure 1 gives the predictive performance (AUC) of each feature selection method (in rows) and classifier (in columns), averaged over the 50-folds/repeats in the cross-validation. Logistic regression models cannot be calculated when the number of predictors is larger than the number of observations, so the nofilter row is blank for this classifier. The large number of predictors also caused multiple computing issues with the neural net classifier, so training this classifier without using any feature selection was not considered. Table 4 gives the highest average AUC for each classifier across the various feature selection methods. Principal component analysis yields lower AUC values for all of the classifying models. Using lincom, the top four classification methods perform well, with AUC ≥ 0.728 (we note that svmr with corr.95 also has an average AUC = 0.728). The standard deviation over the folds/repeats is also given, along with sensitivity, specificity, and false positive rate statistics. Specificity and sensitivity were computed using a 0.5 threshold from the model predicted class probabilities. The AUC standard deviations are fairly similar, while sensitivity and specificity have larger variation. The false positive rates are more variable than the AUC values, and the mean false positive rates are all notably lower (all less than 32%) than the 94% found in the results of the NLST.

**Table 4:** AUC values for classifiers with highest predictive performance (SD taken over the 50 cross-validation testing sets).

	**Feature method**	**AUC**	**SD**	**Sensitivity**	**Specificity**	**False positive rate**	**SD**
**Classifier**							
elasticnet	lincom	0.747	0.111	0.616	0.729	0.271	0.136
svml	lincom	0.745	0.112	0.549	0.765	0.235	0.126
svmpoly	lincom	0.741	0.113	0.569	0.781	0.219	0.132
pls	lincom	0.728	0.111	0.627	0.707	0.293	0.126
svmr	corr.95	0.728	0.106	0.542	0.780	0.220	0.148
gbm	lincom	0.714	0.106	0.596	0.733	0.267	0.140
glmStepAIC	corr.95	0.714	0.110	0.636	0.684	0.316	0.130
nnet	lincom	0.709	0.113	0.620	0.707	0.293	0.143
logistic	corr.95	0.684	0.108	0.600	0.689	0.311	0.116
knn	corr.95	0.676	0.109	0.482	0.738	0.262	0.117
rf	corr.95	0.663	0.124	0.473	0.730	0.270	0.127
bag	lincom	0.658	0.106	0.529	0.702	0.298	0.146

**Figure 2:**
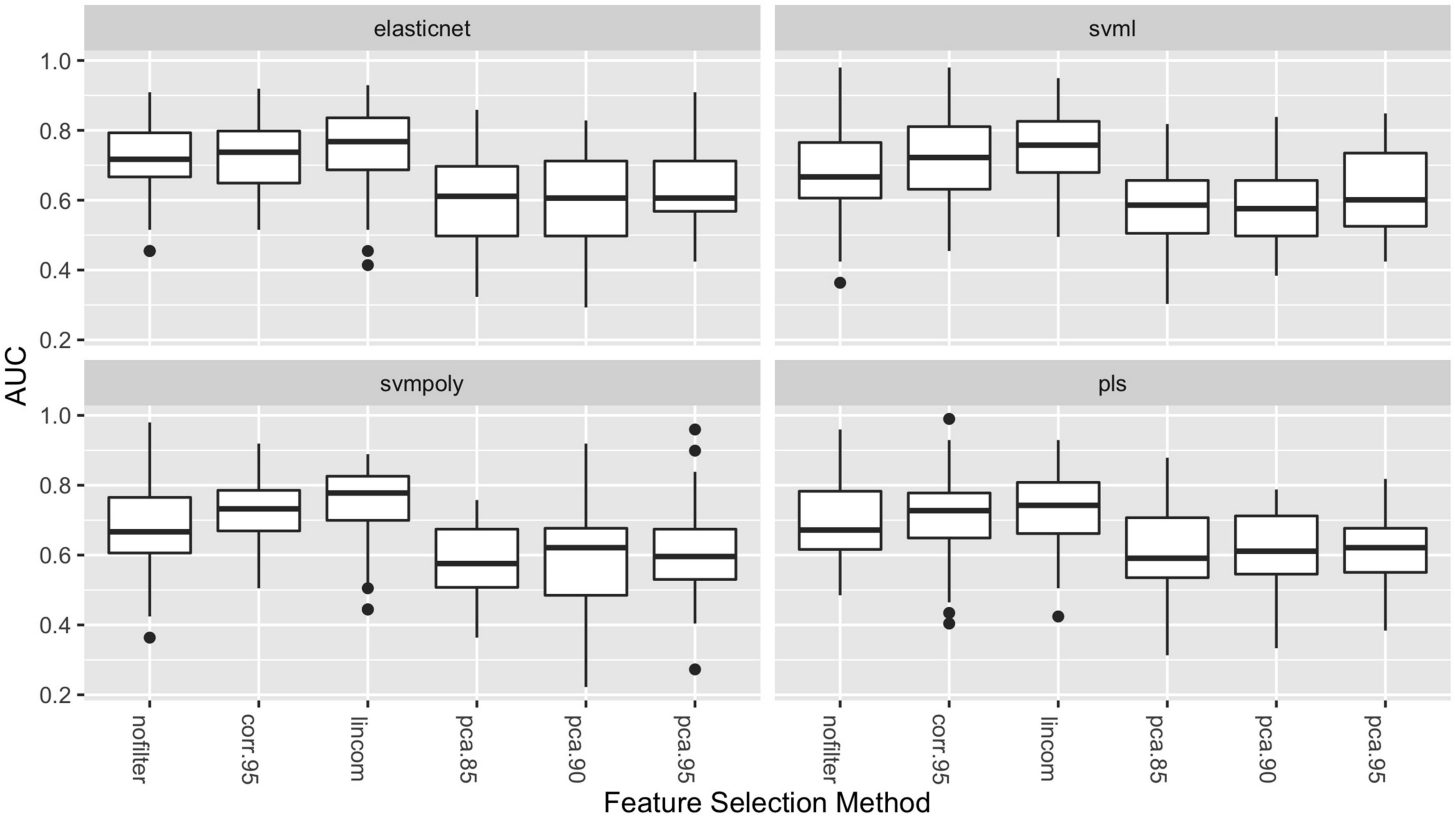
Boxplots of AUC values (over the 50 repeated cross-validation testing sets) for each feature selection method for the four best-performing classifiers.

Figure 2 shows the distribution of the AUC scores for the four best performing classifiers: elasticnet, svml, svmpoly, and pls. Among all feature selection methods, corr.95 and lincom yielded the highest AUC values on average across these four classifiers. The lincom feature selection with the elasticnet classifier has the best overall predictive performance (AUC = 0.747), followed by the svml classifier with the lincom feature selection (AUC = 0.745). As has been observed in other radiomic studies, support vector machines perform well with respect to predictive performance (21).

**Figure 3:**
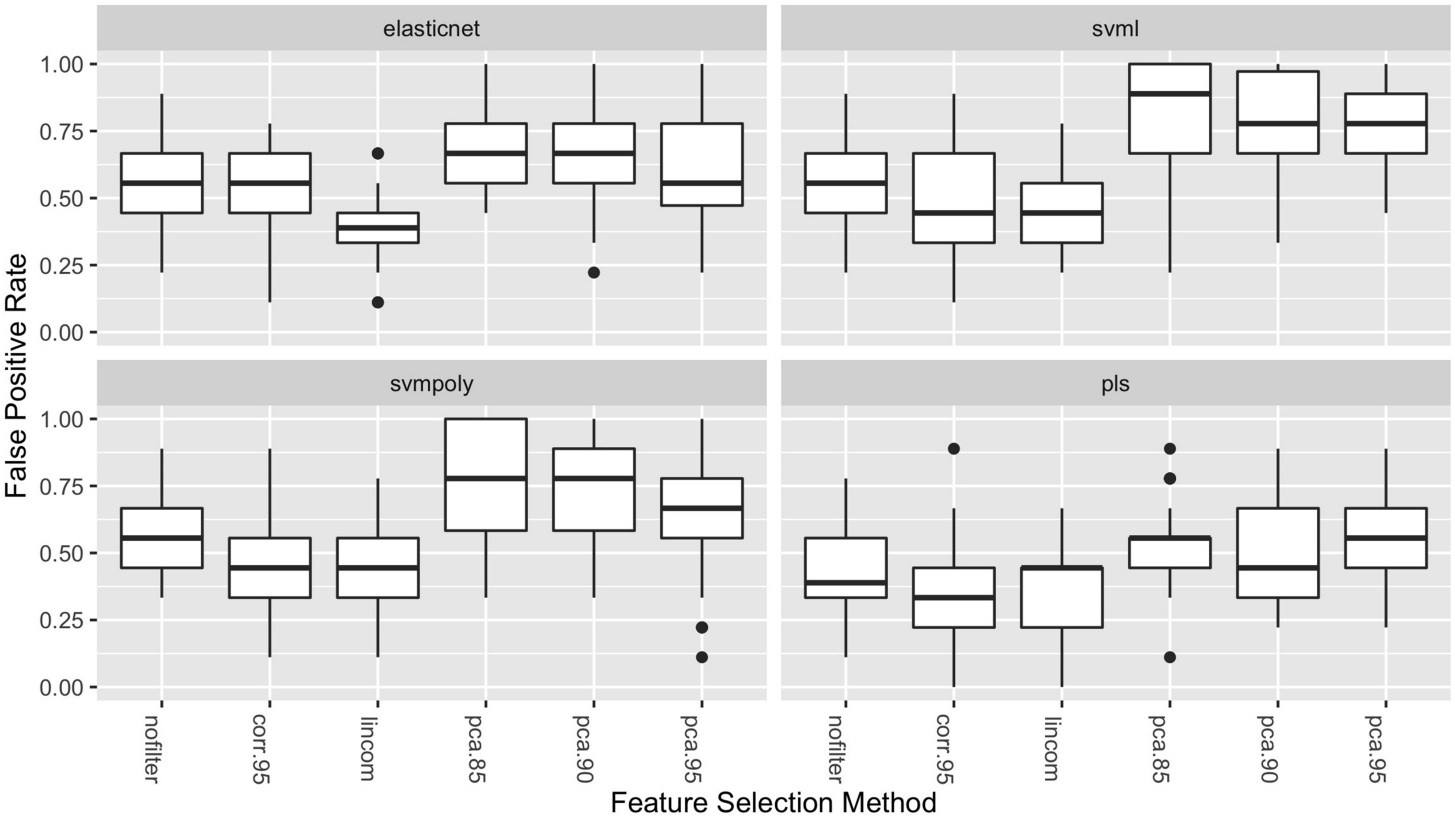
Boxplots of the false positive rates (over the 50 repeated cross-validation testing sets) for each feature selection method for the four best-performing classifiers.

The boxplots in Figure 3 show the distribution of the false positive rates for the four best performing classifiers. These distributions show that the lowest false positive rates were achieved in combination with either the lincom or corr.95 feature selection methods for all four of these classifiers. These two feature selection methods result in both the highest average AUC values and the lowest false positive rates.

**Figure 4:**
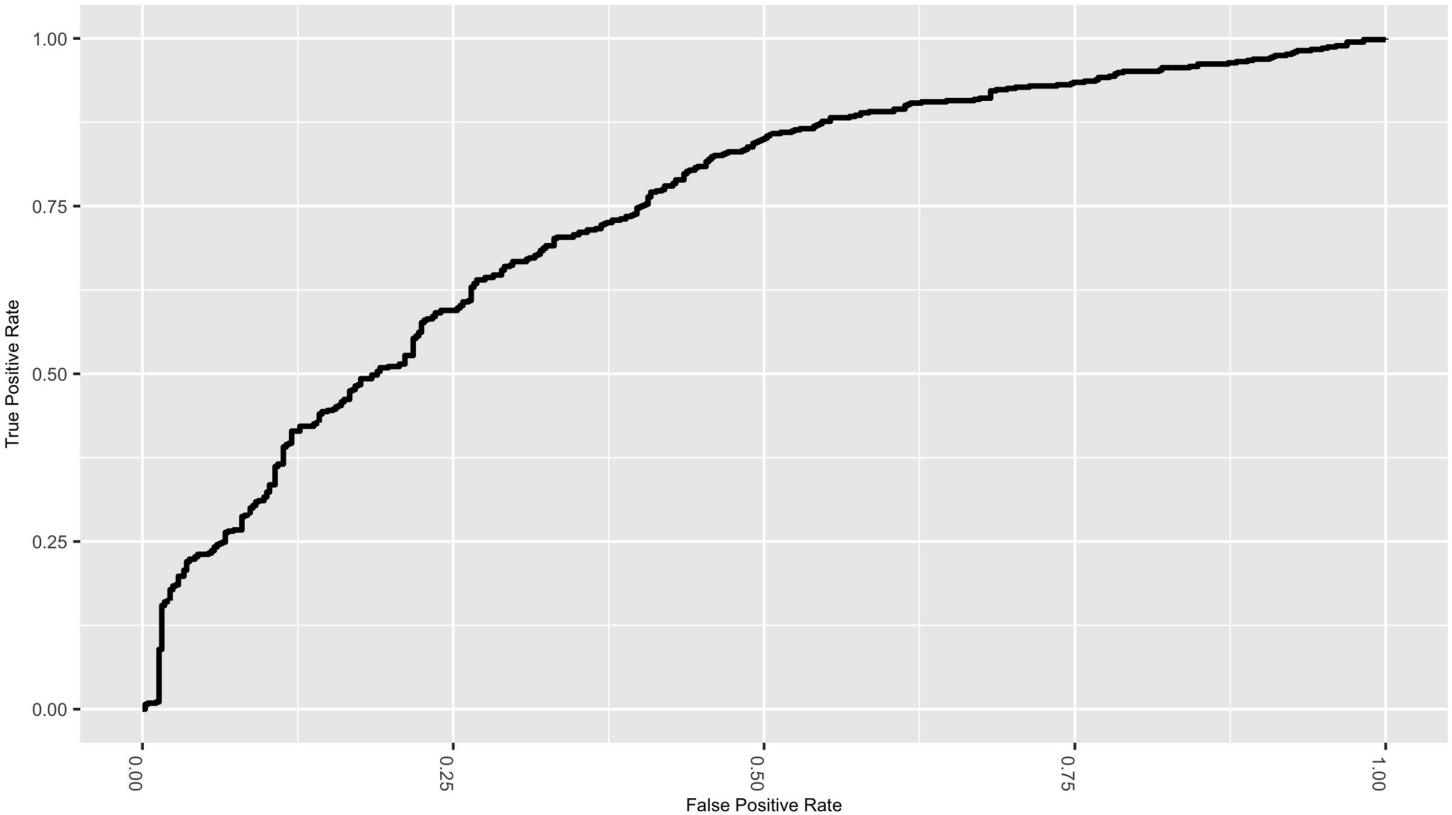
ROC curve for the elastic net classifier with the linear combinations filter.

Figure 4 gives the ROC curve for the best performing classifier/feature selection combination (elasticnet/lincom). Although the NLST did not report false negative rates, the ROC curve displays the tradeoff between specificity and sensitivity. While the classifiers have reduced the false positive rate, the tradeoff is an increase in the false negative rate, which would be estimated to be near 0.38 for this particular classifier. This natural tradeoff between specificity and sensitivity for classifiers would suggest that radiomic methods should not be the sole diagnostic tool in lung cancer diagnosis. However, the reduction of the false positive rate for a non-invasive procedure is a substantial improvement and supports the inclusion of these methods in clinical practice.

## 4. Discussion

While awareness of the benefits of preventative screening for lung cancer has increased in recent years, there is still a need for improved accuracy in nodule classification. Moreover, a high false positive rate for the diagnostic outcome of lung cancer screening remains a major challenge. Nodule characteristics (biomarkers) calculated from CT scans offer the possibility of improved nodule classification through various modeling techniques. Machine learning algorithms have the potential to harness the predictive power in nodule characteristics. However, little work has been done to compare the performance of various machine learning methods used in conjunction with different feature selection methods, especially as they relate to lung cancer tumor diagnosis.

However, models to predict pulmonary nodule status have been developed and evaluated in other studies. Chen et al. extracted 750 imaging features and compared the performance of a support vector machine (SVM) trained with all to an SVM trained with a sequential forward selection of 4 features (2). Leave-one-out cross-validation demonstrated superior accuracy of 84% for the 4-feature model vs. 56% for all features. Alahmari et al. studied the prognostic performance of radiomics features and found the addition of feature changes over time (delta radiomics) to improve AUC performance from 0.773 to 0.822 (25). SVM and random forest models as well as different feature selection algorithms were considered in their analysis. Final results are presented for random forest models and ReliefF feature selection, suggesting that these were the optimal choices, although comparisons to the others were not presented. A computer-aided lung nodule detection system was proposed by Ma et al. (26). In their approach, multiscale nodule and vessel enhancement filters were applied to patient images prior to extracting 979 radiomics features for training of a random forest classifier. Comparisons to other modeling approaches were not made. Uthoff et al. used a set of 922 radiomics features that is an extension of ours with both nodule features and parenchyma features calculated in 25, 50, 75, and 100% bands around the maximal in-plane diameter of the nodule (27). They used k-medoids clustering to select features for training of an artificial neural network. K-medoids feature selection is similar in spirit to the high correlation selection approach we used in that both reduce the number of features by selecting representative ones from those that are similar. Comparisons to other modeling approaches are not presented in their publication.

In this study, we considered the ability of nodule biomarkers to accurately predict malignant/benign status. The elastic net, support vector machines with polynomial and linear kernels, and partial least squares were the most predictive classifiers. When combined with the linear combination and correlation feature selection methods, these four classifiers had AUC values comparable in accuracy to the most predictive models studied in previous radiomic analyses (14, 16, 21). Furthermore, we observed that these classifiers greatly reduced the false positive rate from that given in the NLST results.

The observations from this investigation suggest that classifiers such as support vector machines and elastic net perform well with quantitative imaging biomarkers as their predictors. We also show that the chosen feature selection method will impact model performance, and we recommend using linear combination or a correlation-based reduction method over principal components. Different CT modalities and/or different patient population characteristics may yield different results. In order to recommend a particular model for application in a clinical setting, these results would need to be externally validated.

As as comparison, the two best classifier/feature selection combinations were fit with both the 416 biomarkers, as well as the demographic variables of sex, age, and pack-years (the number of packs smoked per day multiplied by the number of years smoked). Elastic Net with the Linear Combination filter had an average AUC of 0.747 (see Table 4) without the demographic variables included. This number was increased to 0.854 when these variables were added. The Linear Support Vector Machine with the Linear Combination filter had an average AUC of 0.745 without the demographic variables included. This number was increased to 0.820 when these variables were added. This suggests that radiomic features, while having good predictive performance, can be enhanced when other patient characteristics are included in the model.

Taken together, a number of common themes emerge from our present work and the past work of others. First, methods that reduce the number of features prior to model training appear to improve predictive performance. We believe this is especially true in the field of radiomics where large numbers of features tend to be highly correlated. Oftentimes, there are many features that do not provide additional information because they are linear combinations of others and may be removed with a linear combination filter. In addition, radiomics features tend to exhibit strong clustering for which high correlation or k-medoid selection seems to improve prediction even when in the cases of models, like random forests and gradient boosting, that perform automatic feature selection. Second, our work suggests that SVM performs well in the radiomics setting and supports its use by others. Furthermore, we found the commonly used random forest model to have poor performance; whereas, the less commonly used in radiomics—but commonly used in genomics—elastic net model was our top performer. Thus, we encourage consideration and reporting of more than one modeling approach in radiomics research. Finally, there is strong evidence that pulmonary features derived from the parenchyma and that reflect changes over time help with prediction. Likewise, as is the case in many fields, improvements in prediction are often achieved when utilizing subject matter expertise in the development of features and modeling approaches.

## Data Availability Statement

All datasets generated for this study are included in the article/Supplementary Material.

## Author Contributions

All authors listed have made a substantial, direct and intellectual contribution to the work, and approved it for publication.

### Conflict of Interest

The authors declare that the research was conducted in the absence of any commercial or financial relationships that could be construed as a potential conflict of interest.
