# Corrigendum: Machine Learning and Feature Selection Methods for Disease Classification With Application to Lung Cancer Screening Image Data

**DOI:** 10.3389/fonc.2020.00866

**Published:** 2020-06-05

**Authors:** Darcie A. P. Delzell, Sara Magnuson, Tabitha Peter, Michelle Smith, Brian J. Smith

**Affiliations:** ^1^Department of Mathematics and Computer Science, Wheaton College, Wheaton, IL, United States; ^2^Department of Biostatistics, University of Iowa, Iowa City, IA, United States

**Keywords:** radiomics, machine learning, CT image, biomarkers, lung cancer

The data analyzed for this study were generated by Samantha Dilger, Ph.D and Jessica Sieren, Ph.D (Departments of Radiology and Biomedical Engineering, University of Iowa, Iowa City, IA, United States) who control the rights to the data and do not intend for the data to be shared publicly. Accordingly, this data which was included as Supplementary Material in the original article is being removed. In addition, the data were taken from a mix of low and high-dose CT scans, which were incorrectly referred to in the original article as low-dose scans.

The corrections below have been made to the **Methods**, subsection **Dataset**, paragraph 1.

“This retrospective study analyzed data originally taken from 200 CT scans of the lungs of patients at the University of Iowa Hospital. Pathology and radiology reports were reviewed to identify an analysis set of patients who met eligibility criteria of having (a) a solitary lung nodule (5–30 mm) and (b) a malignant nodule confirmed on histopathology or a benign nodule confirmed on histopathology or by size stability for at least 24 months. Manual segmentations were performed by a graduate student trained in medical image analysis in order to define a region of interest (ROI) around each nodule. The ROIs were defined to include amounts of parenchyma approximately proportional to the nodule sizes. Individual ROI voxels were labeled as belonging to either the nodule or the parenchyma, with radiomic features calculated separately for each to produce the complete set of 416 (approximately half nodule and half parenchyma) quantitative imaging biomarkers. These biomarkers measured features such as intensity, shape, and texture of the ROI (15). This study is a secondary analysis of de-identified data originally collected with approval from the University of Iowa institutional review board. Demographic information can be found in Table 1.”

The dataset has been removed from the online Supplementary Material and replaced with R code implementing the feature selection and classification models described in Methods Sections 2.3 and 2.4 of the article. The **Methods** section, subsection **Classifiers and Performance Metrics**, paragraph 2 has been updated to include a reference to the supplementary code as follows:

“The quality of model performance in most machine learning algorithms is dependent upon the choice of various tuning parameters. Some tuning parameters take into account the number of predictors after feature selection. For example, the mtry tuning parameter for rf, which determines the number of candidate variables at each branch, is equal to the square root of the number of predictors. Other tuning parameters were chosen based on standard practice (22, 23). For example, the decay tuning parameter for nnet, which helps prevent overfitting, generally takes the values of 0.1, 0.01, and 0.001. All models were fit using the caret R package (24). Our R code implementing the feature selection and classification models is presented as Supplementary Material.”

The authors apologize for the inclusion of the data in the Supplementary Material and misstatement of “low-dose” CT. We state that these do not change the scientific conclusions of the article in any way. The original article has been updated.

