# Supplementary material for: Machine Learning and Feature Selection Methods for Disease Classification With Application to Lung Cancer Screening Image Data
Source: Front Oncol. 2019 Dec 11;9:1393. doi: 10.3389/fonc.2019.01393 (PMC6917601; doi:10.3389/fonc.2019.01393)
Supplement: Supplementary file 1 [file Presentation_1.PDF]

Supplementary R Software Code

Introduction

This supplement presents R software code implementing the feature selection and classification models described in Sections 2.3 and 2.4 of the accompanying article. The code assumes that the data are stored as a data frame named `rad` containing the following variables.

- Response Variable
  - `BinaryDiagnosis`: factor (No = benign, Yes = malignant)
- Demographic Variables
  - `Age`: numeric
  - `Pack_Years`: numeric
  - `Sex`: factor(F = female, M = male)
- Radiomics Variables: numeric

```
## Load analysis libraries
library(tidyverse)
library(caret)
library(recipes)

## Create analytic data frame without demographic variables
rad_nodemo <- rad %>% select(-Age, -Pack_Years, -Sex)
```

Preprocessing Recipes

```
rec.base <- recipe(x = rad_nodemo) %>%
  update_role(BinaryDiagnosis, new_role = "outcome") %>%
  update_role(-BinaryDiagnosis, new_role = "predictor") %>%
  step_YeoJohnson(all_predictors(), -BinaryDiagnosis) %>%
  step_center(all_predictors(), -BinaryDiagnosis) %>%
  step_scale(all_predictors(), -BinaryDiagnosis)
rec.corr.90 <- rec.base %>% step_corr(all_predictors(), -BinaryDiagnosis, threshold = .90)
rec.corr.95 <- rec.base %>% step_corr(all_predictors(), -BinaryDiagnosis, threshold = .95)
rec.corr.85 <- rec.base %>% step_corr(all_predictors(), -BinaryDiagnosis, threshold = .85)
rec.lincom <- rec.base %>% step_lincomb(all_predictors(), -BinaryDiagnosis)
rec.pca.85 <- rec.base %>% step_pca(all_predictors(), -BinaryDiagnosis, threshold = .85)
rec.pca.90 <- rec.base %>% step_pca(all_predictors(), -BinaryDiagnosis, threshold = .90)
rec.pca.95 <- rec.base %>% step_pca(all_predictors(), -BinaryDiagnosis, threshold = .95)
```

Validation Specification

```
fitControlCV <- trainControl(
  method = "repeatedcv",
```

```
  number = 10,
  repeats = 5,
  classProbs = TRUE,
  summaryFunction = twoClassSummary,
  savePredictions = TRUE
)
```

Logistic Regression

```
set.seed(21)
(clf.logistic.corr.95 <- train(rec.corr.95,
                               data = rad_nodemo,
                               method = "glm",
                               trControl = fitControlCV,
                               metric = "ROC"))
(clf.logistic.lincom <- train(rec.lincom,
                              data = rad_nodemo,
                              method = "glm",
                              trControl = fitControlCV,
                              metric = "ROC"))
(clf.logistic.pca.85 <- train(rec.pca.85,
                              data = rad_nodemo,
                              method = "glm",
                              trControl = fitControlCV,
                              metric = "ROC"))
(clf.logistic.pca.90 <- train(rec.pca.90,
                              data = rad_nodemo,
                              method = "glm",
                              trControl = fitControlCV,
                              metric = "ROC"))
(clf.logistic.pca.95 <- train(rec.pca.95,
                              data = rad_nodemo,
                              method = "glm",
                              trControl = fitControlCV,
                              metric = "ROC"))
```

Partial Least Squares

```
set.seed(21)

plsGrid <- data.frame(ncomp = 1:10)

(clf.pls.basic <- train(rec.base,
                        data = rad_nodemo,
                        tuneGrid = plsGrid,
                        method = "pls",
                        trControl = fitControlCV,
                        metric = "ROC"))
(clf.pls.corr.95 <- train(rec.corr.95,
                          data = rad_nodemo,
```

```
                          tuneGrid = plsGrid,
                          method = "pls",
                          trControl = fitControlCV,
                          metric = "ROC"))
(clf.pls.lincom <- train(rec.lincom,
                         data = rad_nodemo,
                         tuneGrid = plsGrid,
                         method = "pls",
                         trControl = fitControlCV,
                         metric = "ROC"))
(clf.pls.pca.85 <- train(rec.pca.85,
                         data = rad_nodemo,
                         method = "pls",
                         trControl = fitControlCV,
                         metric = "ROC"))
(clf.pls.pca.90 <- train(rec.pca.90,
                         data = rad_nodemo,
                         method = "pls",
                         trControl = fitControlCV,
                         metric = "ROC"))
(clf.pls.pca.95 <- train(rec.pca.95,
                         data = rad_nodemo,
                         method = "pls",
                         trControl = fitControlCV,
                         metric = "ROC"))
```

Elastic Net

```
set.seed(21)

glmnetGrid <- expand.grid(
  lambda = c(0.0001, 0.001, 0.01, 0.1),
  alpha = seq(0.05, 1, by = 0.05)
)

(clf.glmnet.basic <- train(rec.base,
                           data = rad_nodemo,
                           method = "glmnet",
                           tuneGrid = glmnetGrid,
                           trControl = fitControlCV,
                           metric = "ROC"))
(clf.glmnet.corr.95 <- train(rec.corr.95,
                             data = rad_nodemo,
                             method = "glmnet",
                             tuneGrid = glmnetGrid,
                             trControl = fitControlCV,
                             metric = "ROC"))
(clf.glmnet.lincom <- train(rec.lincom,
                            data = rad_nodemo,
                            method = "glmnet",
                            tuneGrid = glmnetGrid,
                            trControl = fitControlCV,
```

```
                             metric = "ROC"))
(clf.glmnet.pca.85 <- train(rec.pca.85,
                             data = rad_nodemo,
                             method = "glmnet",
                             tuneGrid = glmnetGrid,
                             trControl = fitControlCV,
                             metric = "ROC"))
(clf.glmnet.pca.90 <- train(rec.pca.90,
                             data = rad_nodemo,
                             method = "glmnet",
                             tuneGrid = glmnetGrid,
                             trControl = fitControlCV,
                             metric = "ROC"))
(clf.glmnet.pca.95 <- train(rec.pca.95,
                             data = rad_nodemo,
                             method = "glmnet",
                             tuneGrid = glmnetGrid,
                             trControl = fitControlCV,
                             metric = "ROC"))
```

Support Vector Machine (Radial)

```
set.seed(21)

svmrGrid <- expand.grid(
  sigma = c(0.001, 0.005, 0.01, 0.015, 0.02),
  C = c(0.25, 0.5, 1, 1.5, 2, 2.5, 3, 3.5, 4, 4.5, 5, 5.5, 8, 16)
)

(clf.svmr.basic <- train(rec.base,
                          data = rad_nodemo,
                          method = "svmRadial",
                          tuneGrid = svmrGrid,
                          trControl = fitControlCV,
                          metric = "ROC"))
(clf.svmr.corr.95 <- train(rec.corr.95,
                            data = rad_nodemo,
                            method = "svmRadial",
                            tuneGrid = svmrGrid,
                            trControl = fitControlCV,
                            metric = "ROC"))
(clf.svmr.lincom <- train(rec.lincom,
                           data = rad_nodemo,
                           method = "svmRadial",
                           tuneGrid = svmrGrid,
                           trControl = fitControlCV,
                           metric = "ROC"))
(clf.svmr.pca.85 <- train(rec.pca.85,
                           data = rad_nodemo,
                           method = "svmRadial",
                           tuneGrid = svmrGrid,
                           trControl = fitControlCV,
```

```r
                           metric = "ROC"))
(clf.svmr.pca.90 <- train(rec.pca.90,
                          data = rad_nodemo,
                          method = "svmRadial",
                          tuneGrid = svmrGrid,
                          trControl = fitControlCV,
                          metric = "ROC"))
(clf.svmr.pca.95 <- train(rec.pca.95,
                          data = rad_nodemo,
                          method = "svmRadial",
                          tuneGrid = svmrGrid,
                          trControl = fitControlCV,
                          metric = "ROC"))
```

Support Vector Machine (Linear)

```r
set.seed(21)

svmlGrid <- data.frame(C = seq(0.05, 1, by = 0.01))

(clf.svml.basic <- train(rec.base,
                         data = rad_nodemo,
                         method = "svmLinear",
                         tuneGrid = svmlGrid,
                         trControl = fitControlCV,
                         metric = "ROC"))
(clf.svml.corr.95 <- train(rec.corr.95,
                           data = rad_nodemo,
                           method = "svmLinear",
                           tuneGrid = svmlGrid,
                           trControl = fitControlCV,
                           metric = "ROC"))
(clf.svml.lincom <- train(rec.lincom,
                          data = rad_nodemo,
                          method = "svmLinear",
                          tuneGrid = svmlGrid,
                          trControl = fitControlCV,
                          metric = "ROC"))
(clf.svml.pca.85 <- train(rec.pca.85,
                          data = rad_nodemo,
                          method = "svmLinear",
                          tuneGrid = svmlGrid,
                          trControl = fitControlCV,
                          metric = "ROC"))
(clf.svml.pca.90 <- train(rec.pca.90,
                          data = rad_nodemo,
                          method = "svmLinear",
                          tuneGrid = svmlGrid,
                          trControl = fitControlCV,
                          metric = "ROC"))
(clf.svml.pca.95 <- train(rec.pca.95,
                          data = rad_nodemo,
```

```r
                          method = "svmLinear",
                          tuneGrid = svmlGrid,
                          trControl = fitControlCV,
                          metric = "ROC"))
```

Support Vector Machine (Polynomial)

```r
set.seed(21)

svmpolyGrid <- expand.grid(
  degree = 1:3,
  scale = c(0.001, 0.01, 0.1),
  C = c(0.2, 0.25, 0.5, 1, 1.5, 2, 2.5, 3, 3.5, 4, 4.5, 5, 5.5, 6, 8, 16, 32)
)

(clf.svmpoly.basic <-train(rec.base,
                           data = rad_nodemo,
                           method = "svmPoly",
                           tuneGrid = svmpolyGrid,
                           trControl = fitControlCV,
                           metric = "ROC"))
(clf.svmpoly.corr.95 <-train(rec.corr.95,
                             data = rad_nodemo,
                             method = "svmPoly",
                             tuneGrid = svmpolyGrid,
                             trControl = fitControlCV,
                             metric = "ROC"))
(clf.svmpoly.lincom <-train(rec.lincom,
                            data = rad_nodemo,
                            method = "svmPoly",
                            tuneGrid = svmpolyGrid,
                            trControl = fitControlCV,
                            metric = "ROC"))
(clf.svmpoly.pca.85 <-train(rec.pca.85,
                            data = rad_nodemo,
                            method = "svmPoly",
                            tuneGrid =svmpolyGrid,
                            trControl = fitControlCV,
                            metric = "ROC"))
(clf.svmpoly.pca.90 <-train(rec.pca.90,
                            data = rad_nodemo,
                            method = "svmPoly",
                            tuneGrid = svmpolyGrid,
                            trControl = fitControlCV,
                            metric = "ROC"))
(clf.svmpoly.pca.95 <-train(rec.pca.95,
                            data = rad_nodemo,
                            method = "svmPoly",
                            tuneGrid = svmpolyGrid,
                            trControl = fitControlCV,
                            metric = "ROC"))
```

K-Nearest Neighbors

```
set.seed(21)
(clf.knn.basic <- train(rec.base,
                        data = rad_nodemo,
                        method = "knn",
                        trControl = fitControlCV,
                        tuneLength = 75,
                        metric = "ROC"))
(clf.knn.corr.95 <- train(rec.corr.95,
                         data = rad_nodemo,
                         method = "knn",
                         trControl = fitControlCV,
                         tuneLength = 75,
                         metric = "ROC"))
(clf.knn.lincom <- train(rec.lincom,
                         data = rad_nodemo,
                         method = "knn",
                         trControl = fitControlCV,
                         tuneLength = 75,
                         metric = "ROC"))
(clf.knn.pca.85 <-train(rec.pca.85,
                        data = rad_nodemo,
                        method = "knn",
                        trControl = fitControlCV,
                        tuneLength = 75,
                        metric = "ROC"))
(clf.knn.pca.90 <- train(rec.pca.90,
                         data = rad_nodemo,
                         method = "knn",
                         trControl = fitControlCV,
                         tuneLength = 75,
                         metric = "ROC"))
(clf.knn.pca.95 <- train(rec.pca.95,
                         data = rad_nodemo,
                         method = "knn",
                         trControl = fitControlCV,
                         tuneLength = 75,
                         metric = "ROC"))
```

Random Forest

```
set.seed(21)

rfGrid <- data.frame(mtry = 3:21)

(clf.rf.basic <- train(rec.base,
                       data = rad_nodemo,
                       method = "rf",
                       tuneGrid = rfGrid,
                       ntrees = 1000,
                       importance = TRUE,
```

```
                      metric = "ROC",
                      trControl = fitControlCV))
(clf.rf.corr.95 <- train(rec.corr.95,
                        data = rad_nodemo,
                        method = "rf",
                        tuneGrid = rfGrid,
                        ntrees = 1000,
                        importance = TRUE,
                        metric = "ROC",
                        trControl = fitControlCV))
(clf.rf.lincom <- train(rec.lincom,
                       data = rad_nodemo,
                       method = "rf",
                       tuneGrid = rfGrid,
                       ntrees = 1000,
                       importance = TRUE,
                       metric = "ROC",
                       trControl = fitControlCV))
(clf.rf.pca.85 <- train(rec.pca.85,
                       data = rad_nodemo,
                       method = "rf",
                       tuneGrid = rfGrid,
                       ntrees = 1000,
                       importance = TRUE,
                       metric = "ROC",
                       trControl = fitControlCV))
(clf.rf.pca.90 <- train(rec.pca.90,
                       data = rad_nodemo,
                       method = "rf",
                       tuneGrid = rfGrid,
                       ntrees = 1000,
                       importance = TRUE,
                       metric= "ROC",
                       trControl = fitControlCV))
(clf.rf.pca.95 <- train(rec.pca.95,
                       data = rad_nodemo,
                       method = "rf",
                       tuneGrid = rfGrid,
                       ntrees = 1000,
                       importance = TRUE,
                       metric = "ROC",
                       trControl = fitControlCV))
```

Stochastic Gradient Boosting

```
set.seed(21)

gbmGrid <- expand.grid(interaction.depth = c(1, 2),
                       n.trees = seq(50, 200, by = 10),
                       shrinkage = c(0.01, 0.1),
                       n.minobsinnode = seq(5, 40, by = 5))
```

```r
(clf.gbm.basic <- train(rec.base,
                        data = rad_nodemo,
                        method = "gbm",
                        tuneGrid = gbmGrid,
                        trControl = fitControlCV,
                        metric = "ROC"))
(clf.gbm.corr.95 <- train(rec.corr.95,
                          data = rad_nodemo,
                          method = "gbm",
                          tuneGrid = gbmGrid,
                          trControl = fitControlCV,
                          metric = "ROC"))
(clf.gbm.lincom <- train(rec.lincom,
                         data = rad_nodemo,
                         method = "gbm",
                         tuneGrid = gbmGrid,
                         trControl = fitControlCV,
                         metric = "ROC"))
(clf.gbm.pca.85 <- train(rec.pca.85,
                         data = rad_nodemo,
                         method = "gbm",
                         tuneGrid = gbmGrid,
                         trControl = fitControlCV,
                         metric = "ROC"))
(clf.gbm.pca.90 <- train(rec.pca.90,
                         data = rad_nodemo,
                         method = "gbm",
                         tuneGrid = gbmGrid,
                         trControl = fitControlCV,
                         metric = "ROC"))
(clf.gbm.pca.95 <- train(rec.pca.95,
                         data = rad_nodemo,
                         method = "gbm",
                         tuneGrid = gbmGrid,
                         trControl = fitControlCV,
                         metric = "ROC"))
```

Bagged Trees

```r
set.seed(21)
(clf.bag.basic <- train(rec.base,
                        data = rad_nodemo,
                        method = "treebag",
                        trControl = fitControlCV,
                        metric = "ROC"))
(clf.bag.corr.95 <- train(rec.corr.95,
                          data = rad_nodemo,
                          method = "treebag",
                          trControl = fitControlCV,
                          metric = "ROC"))
(clf.bag.lincom <- train(rec.lincom,
                         data = rad_nodemo,
```

```
                          method = "treebag",
                          trControl = fitControlCV,
                          metric = "ROC"))
(clf.bag.pca.85 <- train(rec.pca.85,
                          data = rad_nodemo,
                          method = "treebag",
                          trControl = fitControlCV,
                          metric = "ROC"))
(clf.bag.pca.90 <- train(rec.pca.90,
                          data = rad_nodemo,
                          method = "treebag",
                          trControl = fitControlCV,
                          metric = "ROC"))
(clf.bag.pca.95 <- train(rec.pca.95,
                          data = rad_nodemo,
                          method = "treebag",
                          trControl = fitControlCV,
                          metric = "ROC"))
```

Linear Stepwise Feature Selection

```
set.seed(21)
(clf.glmStepAIC.corr.95 <- train(rec.corr.95,
                                  data = rad_nodemo,
                                  method = "glmStepAIC",
                                  trControl = fitControlCV,
                                  metric = "ROC"))
(clf.glmStepAIC.lincom <- train(rec.lincom,
                                 data = rad_nodemo,
                                 method = "glmStepAIC",
                                 trControl = fitControlCV,
                                 metric = "ROC"))
(clf.glmStepAIC.pca.85 <- train(rec.pca.85,
                                 data = rad_nodemo,
                                 method = "glmStepAIC",
                                 trControl = fitControlCV,
                                 metric = "ROC"))
(clf.glmStepAIC.pca.90 <- train(rec.pca.90,
                                 data = rad_nodemo,
                                 method = "glmStepAIC",
                                 trControl = fitControlCV,
                                 metric = "ROC"))
(clf.glmStepAIC.pca.95 <- train(rec.pca.95,
                                 data = rad_nodemo,
                                 method = "glmStepAIC",
                                 trControl = fitControlCV,
                                 metric = "ROC"))
```

Neural Networks

```
set.seed(21)

nnetGrid <- expand.grid(
  size = 1:10,
  decay = c(0, 1, 2, 0.01, 0.001, 0.0001, 0.00001)
)

(clf.nnet.corr.95 <- train(rec.corr.95,
                           data = rad_nodemo,
                           maxit = 1000,
                           method = "nnet",
                           tuneGrid = nnetGrid,
                           trControl = fitControlCV,
                           metric = "ROC"))
(clf.nnet.lincom <- train(rec.lincom,
                         data = rad_nodemo,
                         maxit = 1000,
                         method = "nnet",
                         tuneGrid = nnetGrid,
                         trControl = fitControlCV,
                         metric = "ROC"))
(clf.nnet.pca.85 <- train(rec.pca.85,
                          data = rad_nodemo,
                          maxit = 1000,
                          method = "nnet",
                          tuneGrid = nnetGrid,
                          trControl = fitControlCV,
                          metric = "ROC"))
(clf.nnet.pca.90 <- train(rec.pca.90,
                          data = rad_nodemo,
                          maxit = 1000,
                          method = "nnet",
                          tuneGrid = nnetGrid,
                          trControl = fitControlCV,
                          metric = "ROC"))
(clf.nnet.pca.95 <- train(rec.pca.95,
                          data = rad_nodemo,
                          maxit = 1000,
                          method = "nnet",
                          tuneGrid = nnetGrid,
                          trControl = fitControlCV,
                          metric = "ROC"))
```
